# Prediction of Biological Motion Perception Performance from Intrinsic Brain Network Regional Efficiency

**DOI:** 10.3389/fnhum.2016.00552

**Published:** 2016-11-02

**Authors:** Zengjian Wang, Delong Zhang, Bishan Liang, Song Chang, Jinghua Pan, Ruiwang Huang, Ming Liu

**Affiliations:** ^1^Key Laboratory of Mental Health and Cognitive Science of Guangdong Province, Center for the Study of Applied Psychology, School of Psychology, South China Normal UniversityGuangzhou, China; ^2^Guangdong Polytechnic Normal UniversityGuangzhou, China; ^3^Guangzhou LibraryGuangzhou, China

**Keywords:** biological motion, resting-state network, network efficiency, multiple linear regression model, brain-behavior analysis

## Abstract

Biological motion perception (BMP) refers to the ability to perceive the moving form of a human figure from a limited amount of stimuli, such as from a few point lights located on the joints of a moving body. BMP is commonplace and important, but there is great inter-individual variability in this ability. This study used multiple regression model analysis to explore the association between BMP performance and intrinsic brain activity, in order to investigate the neural substrates underlying inter-individual variability of BMP performance. The resting-state functional magnetic resonance imaging (rs-fMRI) and BMP performance data were collected from 24 healthy participants, for whom intrinsic brain networks were constructed, and a graph-based network efficiency metric was measured. Then, a multiple linear regression model was used to explore the association between network regional efficiency and BMP performance. We found that the local and global network efficiency of many regions was significantly correlated with BMP performance. Further analysis showed that the local efficiency rather than global efficiency could be used to explain most of the BMP inter-individual variability, and the regions involved were predominately located in the Default Mode Network (DMN). Additionally, discrimination analysis showed that the local efficiency of certain regions such as the thalamus could be used to classify BMP performance across participants. Notably, the association pattern between network nodal efficiency and BMP was different from the association pattern of static directional/gender information perception. Overall, these findings show that intrinsic brain network efficiency may be considered a neural factor that explains BMP inter-individual variability.

## Introduction

Biological motion perception (BMP) is the ability of the visual system to perceive movement from a limited amount of visual stimuli (Blake and Shiffrar, 2007). Individuals can effortlessly extract social information from human movements, even from only a small selection of cues indicating movement (Johansson, 1973; Troje, 2002). Johansson (1973) showed that when human motion is represented by point light displays (PLD) that consist of 10–13 points of light attached to the major joints of the body, human observers could easily identify the moving human form despite only seeing the moving lights. BMP with PLDs is still robust when the local motions are masked by noise (Cutting et al., 1988; Bertenthal and Pinto, 1994), and even inversion (Pavlova and Sokolov, 2000). BMP with point-light displays can also convey a range of socially relevant information, including gender (Mather and Murdoch, 1994), affect (Pollick et al., 2001), personality traits such as trustworthiness (Heberlein et al., 2004), and identity (Troje et al., 2005; Jokisch et al., 2006). BMP has high inter-individual variability based on many different factors such as development conditions (Pavlova et al., 2001; Carter and Pelphrey, 2006), gender effect (Anderson et al., 2013; Pavlova et al., 2014), social cognition and motor imagery abilities (Miller and Saygin, 2013). In a recent study, Gilaie-Dotan et al. (2013) found that the neuroanatomical structures of the posterior superior temporal sulcus and ventral medial prefrontal cortex were linked with biological motion detection performance. However, the underlying neural substrate is still not fully understood.

A number of factors, such as aging (Pilz et al., 2010; Legault et al., 2012), experience (Grossman et al., 2004; Calvo-Merino et al., 2010; Hohmann et al., 2011), and certain diseases (Pavlova et al., 2006; Klin et al., 2009) have been shown to influence individual BMP performance. Previous studies have found that these above factors could also modulate individuals' spontaneous brain activity (Lewis et al., 2009; Dosenbach et al., 2010; Rosazza and Minati, 2011; Taubert et al., 2011). Additionally, spontaneous brain activity could also predict subsequent behavior and mental states (Greicius et al., 2003; Fox et al., 2006; Boly et al., 2007; Northoff et al., 2010). For example, Dosenbach et al. (2010) used functional connectivity of spontaneous brain activity to predict individual brain maturity; Wei et al. (2012) found that spontaneous neuronal activity of the left middle temporal gyrus could predict conceptual processing capacity; and Hashmi et al. (2014) found that spontaneous functional network architecture could predict psychologically mediated analgesia related to treatment in chronic knee pain patients. These results suggest that spontaneous brain activity may be a “source” that is not only modified by traces of past brain activity but also influences present and future brain activity (Hasler and Northoff, 2011; Sadaghiani and Kleinschmidt, 2013; Gess et al., 2014). Thus, the exploration of spontaneous brain activity provides potential opportunities to investigate the neural substrate underlying inter-individual variability in BMP.

In particular, many studies have shown that spontaneous brain activity is interconnected within a network, which can be depicted using graph-based network analysis (Bullmore and Sporns, 2009; Huang et al., 2012). Brain network properties are shown to be largely responsible for cognitive performance, such as working memory (Stevens et al., 2012), intellectual ability (van den Heuvel et al., 2009), and the mediated analgesia effect (Hashmi et al., 2014). These observations provide evidence that the intrinsic topological organization of brain activity determines the actual detailed properties characteristic of perceptual and cognitive processes.

This study attempted to explore the link between the topological organization of the intrinsic brain network and BMP performance. For this purpose, resting-state functional magnetic resonance imaging (rs-fMRI) data were collected from 24 healthy students, and a functional brain network was constructed for each individual participant. We modeled network efficiency (i.e., the global efficiency and local efficiency) based on graph-based models to quantify the topological organization of the brain network and applied multiple linear regression methods to explore the link between network efficiency and BMP performance.

## Materials and methods

### Participants

A total of 24 healthy, right-handed participants (11 males, mean age of 20.63 ± 3.20 years) were recruited from South China Normal University, Guangzhou, China. All of them had normal or corrected-to-normal vision, and none had a history of neurological or psychiatric disease or head injury or used medication for anxiety or depression. The protocol was approved by the Research Ethics Review Board of South China Normal University, and written informed consent was provided by each participant before the experiment.

### Image acquisition

All MRI data were obtained on a 3 T Siemens Trio Tim MR scanner with a 12-channel phased array head coil at South China Normal University. The fMRI data were acquired using a gradient-echo echo-planar imaging (EPI) sequence with the following parameters: *TR* = 2000 ms, *TE* = 30 ms, flip angle = 90°, data matrix = 64 × 64, field of view (FOV) = 224 × 224 mm^2^, slice thickness/inter-slice gap = 3.5/0.8 mm, and 32 axial slices covering the whole brain. In total, 240 volumes were obtained, and the acquisition time was approximately 8 min for the rs-fMRI scan. In addition, we also obtained high-resolution brain structural images by using a T1-weighted 3D MP-RAGE sequence with the following parameters: *TR* = 1900 ms, *TE* = 2.52 ms, flip angle = 9°, data matrix = 256 × 256, FOV = 230 × 230 mm^2^, thickness = 1.0 mm, and 176 sagittal slices covering the whole brain. For each participant, both the rs-fMRI data and the brain structural images were acquired in the same session.

### Behavioral experiment

#### Experimental material

In this study, the BMP performance of each individual participant was assessed using FASTSTONE software (http://www.faststone.org/FSCaptureDetail.htm) on the well-known point-light biological motion stimuli captured from the BIO MOTION LAB (http://www.biomotionlab.ca/Demos/BMLwalker.html). More detailed information has been described in previous studies (Troje, 2002; Perry et al., 2010; Saunders et al., 2010). The 15 virtual markers were located at the joints of the ankles, knees, hips, wrists, elbows, and shoulders, as well as at the center of the pelvis, on the sternum, and on the center of the head. The experimental stimuli included two independent dimensions of biological motion (i.e., gender and walking direction) and no other masks were used. Stationary walking (as on a treadmill) at normal speed in a frontal view (for gender perception) or profile view (for direction perception) was presented.

**Figure 1:**
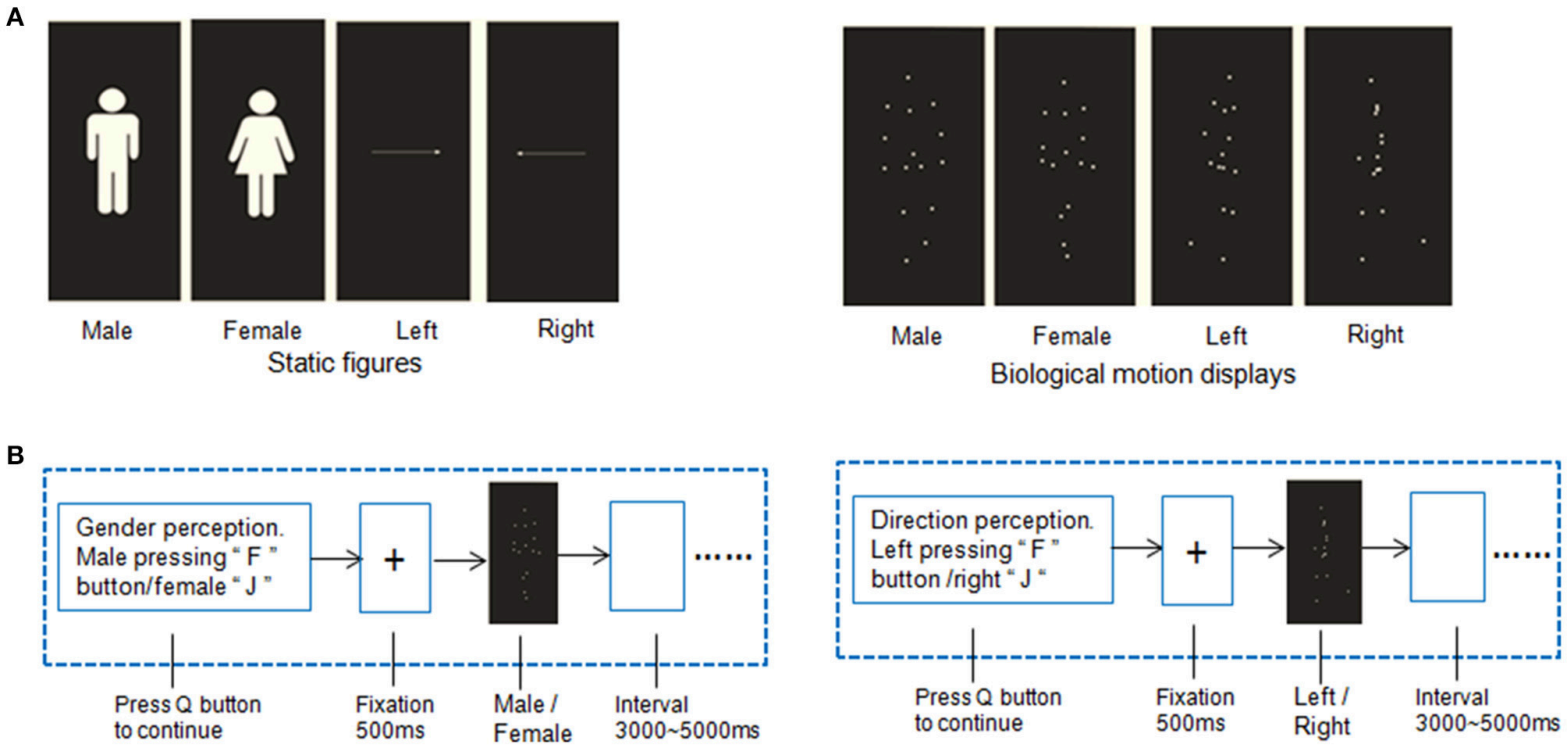
**Example of stimuli and experimental procedures. (A)** Point-light displays of biological motion and static pictures with static gender and directional information. The point-light biological motion stimuli were modified from the BioMotionLab (http://www.biomotionlab.ca/Demos/BMLwalker.html). **(B)** Stimulus paradigm of gender perception of biological motion and static gender perception.

The gender dimension was set in terms of the linear discriminant function that could separate the male and female walkers (Troje, 2002). Maleness was generated by adding to the average walker a vector pointing in the direction of this discriminant function. The length of this vector was set to represent a rather exaggerated male walking style. For a female walker, the same vector was subtracted from the average walker. For the direction dimension, the walkers were facing left or right. The other properties (i.e., heavy/light, nervous/relaxed, and happy/sad) were controlled in average values (Figure 1A). Both dimensions were manipulated independently. For example, a PLD could represent both a woman walking toward the observer and a walker facing left.

All of the stimuli were presented as video clips, and each stimulus presentation lasted 3000 ms. The clips were presented on a CRT monitor 70 cm away from the participant's eyes. In addition, two types of static figures with the same gender/directional information were used as the control condition. The example videos of the PLDs were in the Supplementary Materials.

#### Experimental procedure

The stimuli were presented as 4 blocks (Figure 1B) in an AABB design paradigm. The biological motion gender/direction blocks and static picture gender /direction blocks were balanced across participants, i.e., the biological motion blocks were presented followed by the static figure blocks or vice versa. A total of 30 trials were included in each block. Before each block, a short slide instruction about the following task was presented, telling the subjects to judge the gender or direction of the human figures or static pictures. The participants were asked to press the “Q” button to continue once they understood the instructions.

In the biological motion tasks, the gender and direction perception blocks were also balanced between subjects in AB design paradigm. Each trial in a block started with a fixation cross appearing for 500 ms on the center of the screen, after which the point-light stimulus appeared for 3000 ms. During this period, the participants were instructed to make a decision about the point-light information. For example, in the biological motion gender perception task, participants were asked to press “F” button for male figures and female “J”; in the direction blocks, press “F” button for the left walking direction and right “J.” A complete practice manipulation was implemented to ensure the participants fully understood and were proficient at the tasks.

The control experiment was the same as the main experiment except for the stimuli (i.e., static figures). The stimulus presentation and control experiment were performed using E-Prime (http://www.pstnet.com/eprime.cfm). Only 16 participants completed the control task due to time conflicts. All the trials were completed 1 month following their fMRI scan.

### Brain-behavior analysis

#### Behavioral data analysis

The inverse efficiency (IE) measure (i.e., the average response time of correct trials divided by their accuracies) was used to characterize behavioral performance in biological perception and to correct for the speed-accuracy trade-off effect (Townsend and Ashby, 1983; Falter et al., 2006; Chica et al., 2011; Wei et al., 2012). For the IE calculation, we extracted the average response time and the accuracy ratio in each condition for each individual participant. The accuracy was the ratio between the number of correct responses and the total number of trials (Wei et al., 2012). Then, the average response time of correct trials was divided by the related accuracy ratio to obtain the IE index.

#### Rs-fMRI data preprocessing

The rs-fMRI data preprocessing was performed with the GRETNA toolbox (http://www.nitrc.org/projects/gretna/) based on SPM8 software (http://www.fil.ion.ucl.ac.uk/spm/software/spm8/). After removal of the first 5 volumes, the functional images were first corrected for time offsets between slices and geometrical displacements due to head movement. None of the participants was excluded based on the criterion of a displacement <1 mm in any plane or <1° in any direction. All of the corrected functional data were then spatially normalized to the Montreal Neurological Institute (MNI) space using an optimal 12-parameter affine transformation and nonlinear deformations. Then, the normalized rs-fMRI data were resampled to a 3-mm isotropic resolution and smoothed using a 4-mm isotropic kernel and further temporally band-pass filtered (0.01–0.08 Hz) to reduce the effects of low-frequency drift and high-frequency physiological noise. Finally, the linear trend was also removed, and several nuisance signals were regressed out from each voxel's time series, including 24-parameter head-motion profiles (Friston et al., 1996; Yan et al., 2013), mean white matter (WM) and cerebrospinal fluid (CSF) time series.

#### Brain network construction

**Figure 2:**
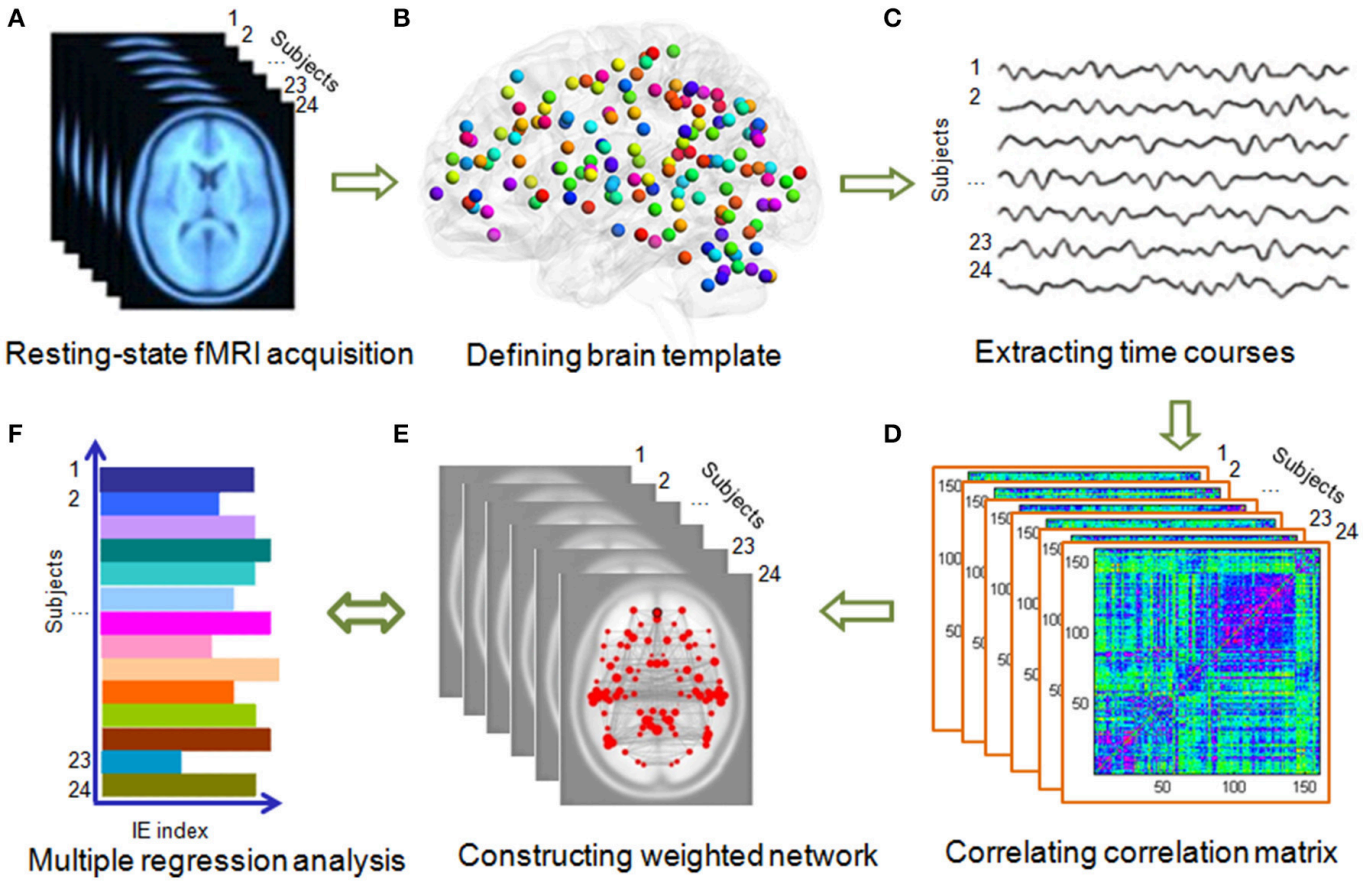
**Flowchart of the network construction. (A)** Acquiring resting-state fMRI data. All the fMRI data were preprocessed using SPM8 software (http://www.fil.ion.ucl.ac.uk/spm/software/spm8/). **(B)** Two brain templates, Dosenbach-160 and AAL-90 atlas were used. **(C)** Time courses extraction from each brain region. **(D)** Computing Pearson correlation of each region with the other regions and transformed it into fisher z value. **(E)** Constructing weighted networks using GRETNA (Wang et al., 2015). **(F)** Using multiple regression model to explore the association between global and local network efficiency with the IE (i.e., the average response time of correct trials was divided by the related accuracy ratio) of gender and direction perception from biological motion and static pictures.

The functional weighted networks were constructed with the nodes corresponding to brain regions and the edges to inter-nodal functional connectivity for each participant. Figure 2 shows the whole process of network construction. A functional template (i.e., 160 regions of interest) from a previous meta-analysis study (Dosenbach et al., 2010) was used to define the nodes of the functional network. The template covers the cerebral cortex, subcortical structures, and the cerebellum, and has been widely used in previous studies (Xue et al., 2011; Hwang et al., 2013; Shen et al., 2015). To define the edge weight of the brain weighted network, we extracted the time series of all voxels within each node and then averaged them to obtain the mean time series. Finally, a 160 × 160 correlation matrix was obtained by calculating Pearson's correlation coefficients among these time series. The analysis was restricted to positive connectivity within network due to the ambiguous interpretation of negative functional connections (Murphy et al., 2009; Weissenbacher et al., 2009). Furthermore, there is still no consensus about whether the negative correlations are artificially induced by global signal regression (Murphy et al., 2009) or if they have biological origins (Chai et al., 2011).

#### Brain network analysis

The functional weighted networks were further fed into the graph-based network analyses. The networks differed in the number of edges (i.e., correlation matrix) (Wen et al., 2011; Shen et al., 2015). Thus, we applied a range of sparsity thresholds, defined as the fraction of the total number of edges remaining in a network, so every graph had the same number of edges (Watts and Strogatz, 1998; Wen et al., 2011; Shen et al., 2015; Suo et al., 2015). The minimum sparsity was set so that the averaged node degree of the network with threshold was 2log(*N*), where *N* was the number of nodes, and the small-worldness scalar of the network was >1.1 (Wen et al., 2011; Shen et al., 2015; Suo et al., 2015). This thresholding strategy produced networks that could be used to estimate small-worldness with sparse properties and the minimum possible number of spurious edges (Wen et al., 2011; Shen et al., 2015; Suo et al., 2015). The subsequent network analyses were repeatedly performed in the small-world regime of 0.03–0.51 in 0.02 increments, which was based on the series of weighted connectivity matrices for each participant.

We used six global parameters, the clustering coefficient (*C*_*w*_), characteristic path length (*L*_*w*_), normalized weighted clustering coefficient (γ), normalized weighted characteristic path length (λ), global efficiency (*E*_*glob*_), and local efficiency (*E*_*loc*_), to characterize the global properties of the brain's functional networks. The above global properties have been widely used in previous studies (Liu et al., 2008; Zhao et al., 2012; Jiang et al., 2013; Wang et al., 2014), and have been defined in Rubinov and Sporns (2010).

##### Global efficiency

Global efficiency is a measure of a network's capacity for parallel information transfer between nodes via multiple series of edges. Mathematically, the global efficiency for a network *G* is defined as:

(1) $$\begin{array}{l} E_{glob}\left(G\right)\;=\;\frac{1}{N\left(N-1\right)}\underset{i\neq \;j\in G}{\sum }\frac{1}{d_{ij}}, \end{array}$$

where *d*_*ij*_ is the shortest path length between node *i* and *j* in *G* and is calculated as the smallest sum of edge lengths among all of the possible paths from node *i* to node *j*.

The global efficiency of a given node, i.e., nodal global efficiency, is defined as:

(2) $$\begin{array}{l} E_{glob}^{nodal}\left(G,i\right)\;=\;\frac{1}{\left(N-1\right)}\underset{i\neq \;j\in G}{\sum }\frac{1}{d_{ij}^{w}}, \end{array}$$

where *N* is the number of nodes in the network *G*. $d_{ij}^{w}$ is the shortest path, in terms of weighted distance, between nodes *i* and *j*. $d_{ij}^{w}\;=\;\frac{d_{ij}}{w_{ij}}$, where *w*_*ij*_ is the connection strength between nodes *i* and *j*.

##### Local efficiency

The local efficiency was calculated as the mean of the local efficiencies across all nodes within a network. The local efficiency of *G* is defined as:

(3) $$\begin{array}{l} E_{loc}\left(G\right)\;=\;\frac{1}{N}\underset{i\in \;G}{\sum }E_{glob}\left(G_{i}\right), \end{array}$$

where *E*_*glob*_(*G*_*i*_) is the global efficiency of *G*_*i*_, the sub-graph composed of the neighbors of node *i*. In parallel, the local efficiency of a given node, i.e., nodal local efficiency, is defined as:

(4) $$\begin{array}{l} E_{loc}^{nodal}(G,i)\;=\;\frac{1}{N_{G_{i}}(N_{G_{i}}-1)}\sum_{k\neq \;j\in \;G_{i}}(\frac{1}{d_{jk}^{w}}w_{ij}w_{ik}{)}^{1/3}, \end{array}$$

where *N_G_i__* is the number of nodes in the subgraph *G*_*i*_ consisting of all the neighbors of *i*. $d_{jk}^{w}$ is the shortest path, in terms of weighted distance, between nodes *j* and *k*. *w*_*ij*_ and *w*_*ik*_ are the connection strength between nodes *i* and *j*, and *i* and *k*, respectively.

##### Network characterization

The clustering coefficient, *C_w_*, is defined as:

(5) $$C_{w}=\frac{1}{N}\sum_{i\in N}\frac{{\sum_{j,h\in N}\left(W_{ij}W_{ih}W_{jh}\right)}^{1/3}}{K_{i}(K_{i}-1)},$$

where *N*_*ij*_ is the weight between node *i* and *j* in a network, and *K*_*i*_ is the degree of node *i*. *C*_*w*_ is the mean of the weighted clustering coefficients of all nodes in a network. It indicates the extent of local interconnectivity or cliquishness in a network. The characteristic path length *L*_*w*_ is defined as:

(6) $$\begin{array}{l} L_{w}=\frac{1}{1/\left(N\left(N-1\right)\right)\overset{N}{\underset{i\;=\;1}{\sum }}\overset{N}{\underset{j\neq i}{\sum }}1/L_{ij}} \end{array}$$

where *L*_*ij*_ is the characteristic path length between nodes *i* and *j*. It measures a harmonic mean length between pairs and quantifies the ability for information propagation in parallel.

The small-world properties of the network were characterized by the normalized clustering coefficient (γ = $C_{w}^{real}/C_{w}^{rand}$) and the normalized characteristic path length (λ = $L_{w}^{real}/L_{w}^{rand}$) (Watts and Strogatz, 1998), where $C_{w}^{rand}$ and $L_{w}^{rand}$ are the averaged weighted clustering coefficient and characteristic path length of 100 matched random networks that keep the same number of nodes, edges, and degree distributions as the actual network. Typically, a small-world network should meet the following criteria: γ ≫ 1 and λ ≈ 1 (Watts and Strogatz, 1998).

#### Network-behavior association analysis

A multiple linear regression with least squares estimation was used to explore the association between BMP performance (i.e., the inverse efficiency, the dependent variable) and the network efficiency (i.e., the global and local efficiency, the independent variables). To this end, the integrated value under all of the sparsities of the selected metrics was calculated for each participant. Then, the metrics of each node was divided by the averaged values over all nodes within each subject for normalization. The BMP performance was characterized using the IE metric, which was calculated for the performance on the two conditions: direction and gender discrimination. Next, a multiple linear regression model was applied to capture the link between the network global and local efficiency and the BMP performance. For our calculations, a feature selection step was used to reduce the data dimension. After the feature selection step, the significant correlation between global and local efficiency of those nodes, and the BMP performance (all *p* < 0.05, Pearson correlation, uncorrected) were entered into a multiple linear regression analysis. The feature selection procedure has been used in our previous work (Zhang et al., 2015).

Similarly, we performed a multiple regression analysis between the global and local efficiency values, and the static picture perception performance (i.e., Static-Direction and Static-Gender).

#### Discrimination analysis

All participants were sorted by the inverse efficiency (i.e., IE index, the average response time of correct trials divided by their accuracies), and then divided into high and low groups at the median split of the IE values (Hashmi et al., 2014). We then implemented the receiver operating characteristic (ROC) analysis to explore whether the network efficiency could clearly distinguish the high inverse efficiency group from low inverse efficiency group. The ROC curve, which is widely used in medical science (Missonnier et al., 2007; Chen et al., 2010; Liu and Zhou, 2013), is a fundamental plot in signal detection theory (Cardillo, 2008). More specifically, the ROC is a scatter plot showing the relationship between false alarm rates and correct rates, and describes the relationship between the underlying distribution of the places where signals were absent and places where signals were present. This analysis was performed using public MATLAB codes (http://www.mathworks.com/matlabcentral/fileexchange/19950; by Giuseppe Cardillo).

### Validation analysis

First, the results of the multiple linear regression analysis between the network efficiency and BMP performance were validated. For this purpose, we shuffled the order of the IE index across the participants to disorganize their correspondences with the network properties. Then, the multiple linear regression procedure was repeated using the selected global and local efficiency and the random IE index. Second, this study also explored whether the network efficiency of those regions that did not have a high correlation with BMP performance could also be used to explain the inter-individual variability of BMP performance. The same number of un-correlated regions with the correlated regions were randomly selected and used as the independent variables in the regression model. Finally, all of the main findings of the brain-behavior analysis with the network of the Dosenbach-160 template were validated using the network constructed with the Automated Anatomical Labeling brain template of 90 cerebral regions (AAL-90) (Tzourio-Mazoyer et al., 2002).

## Results

### Behavioral performance

**Figure 3:**
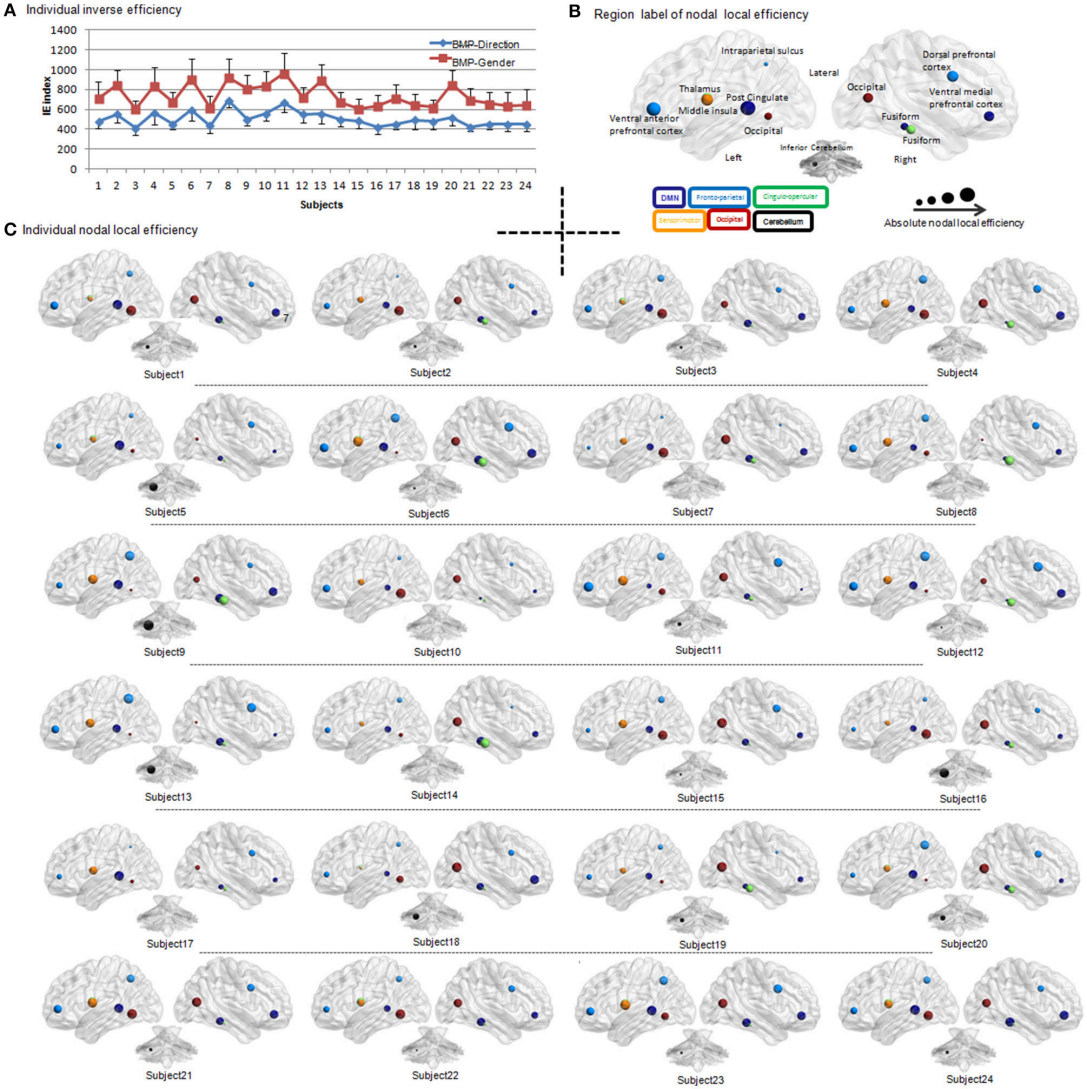
**Individual IE of biological motion perception and individual BMP-Direction correlated nodal local efficiency. (A)** IE of biological motion perception from each participant. The red square indicates the individual's IE (i.e., the average response time of correct trials divided by their accuracies) of gender perception from biological motion; the blue diamond indicates the individual's inverse efficiency of direction perception of biological motion. The error bar is the standard deviation. **(B)** Region label of nodal local efficiency. The regions of which nodal local efficiency showed significant Pearson correlation (uncorrected, *p* < 0.05) with IE of BMP-Direction were showed in different size and colors. The colors indicate the sub-network involved. The size of the spheres is the absolute value of nodal local efficiency. **(C)** Individual BMP-Direction correlated nodal local efficiency. Abbreviations: BMP, biological motion perception; BMP-Direction, direction perception of biological motion; BMP-Gender, gender perception of biological motion.

The accuracy ratios of the two BMP tasks (i.e., BMP-Direction and BMP-Gender) were high (both over 95%). The mean response times, accuracies, and inverse efficiencies (i.e., IE index, the average response time of correct trials divided by their accuracies) of the tasks are shown in Table 1. The inverse efficiency from each participant is shown in Figure 3A. When subjects were divided into two groups (i.e., the high and low IE index groups) based on a median split of their IE index, two sample *t*-test showed significant inter-individual variability on perception task performance [BMP-Direction, *t*_(22)_ = 6.14, *p* < 0.0001; BMP-Gender, *t*_(22)_ = 6.08, *p* < 0.0001].

**Table 1:** **Behavior performance of the participants in this study**.

	**Response time (ms) Mean (*SD*)**	**Accuracy ration Mean (*SD*)**	**Inverse efficiency Mean (*SD*)**
Gender^a^	753.40 (138.10)	0.95 (0.03)	794.38 (147.05)
Direction^a^	505.63 (74.11)	0.96 (0.02)	533.35 (85.93)
Gender^b^	452.46 (85.64)	0.96 (0.03)	470.07 (82.93)
Direction^b^	355.83 (55.09)	0.99 (0.02)	360.71 (52.26)

a *Biological motion information*.

b *Static information*.

### Network topological organization

**Figure 4:**
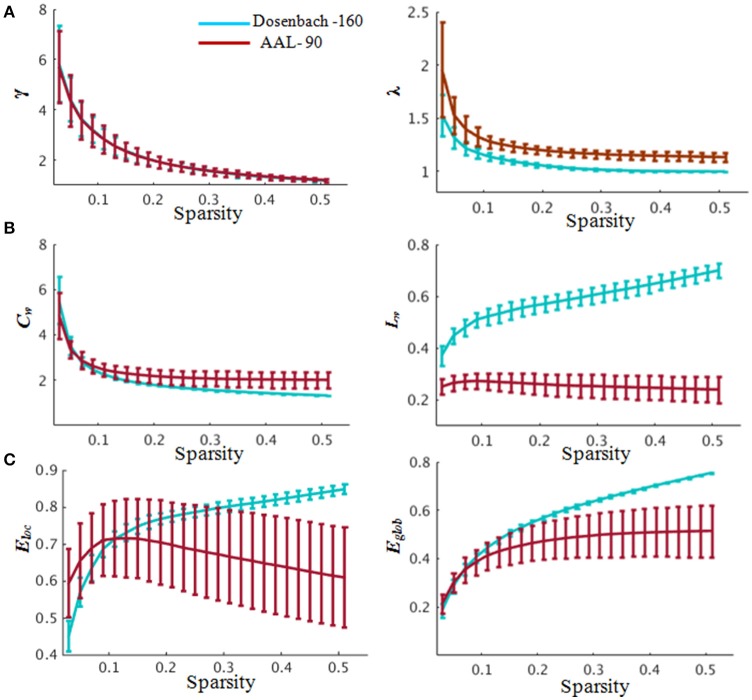
**Global properties of the whole-brain functional network. (A)** Small-worldness properties. In the range of 0.03≤ sparsity ≤ 0.52, the functional networks exhibited γ > 1 and λ ≈ 1, indicating prominent small-world properties of the brain functional networks corresponding to the Dosenbach-160 and AAL-90 brain templates. **(B)** Clustering coefficient (left) and characteristic path length (right) changing with sparsities. **(C)** Global and local efficiency changing with sparsities. Blue indicates network constructed based on Dosenbach-160 and red based on AAL-90 brain template. Abbreviations: γ, normalized weighted clustering coefficient; λ, normalized weighted characteristic path length; *C*_*w*_, weighted clustering coefficient; *L*_*w*_, weighted characteristic path length; *E*_*glob*_, global efficiency, *E*_*loc*_, local efficiency. Error bars correspond to the standard error.

The brain functional weighted networks satisfy small-world organization (λ ≈ 1 and γ > 1) (Figure 4). However, the global and local efficiency of whole brain were not significantly correlated with BMP performance (Pearson correlation, BMP-Direction: global efficiency, *r* = 0.04, *p* = 0.84; local efficiency: *r* = 0.22, *p* = 0.31; BMP-Gender: global efficiency, *r* = 0.09, *p* = 0.67; local efficiency, *r* = 0.27, *p* = 0.21).

### Multiple linear regressions

#### Biological motion perception

In order to reduce the data dimension, the feature selection procedure was applied. In the feature selection procedure, we first computed the Pearson correlation coefficients between the nodal network efficiency (nodal global and local efficiency) with the behavior performance (BMP and static pictures perception performance). Then we selected the significantly correlated regions with threshold of *p* < 0.05 as independent variables for our multiple regression analysis.

**Figure 5:**
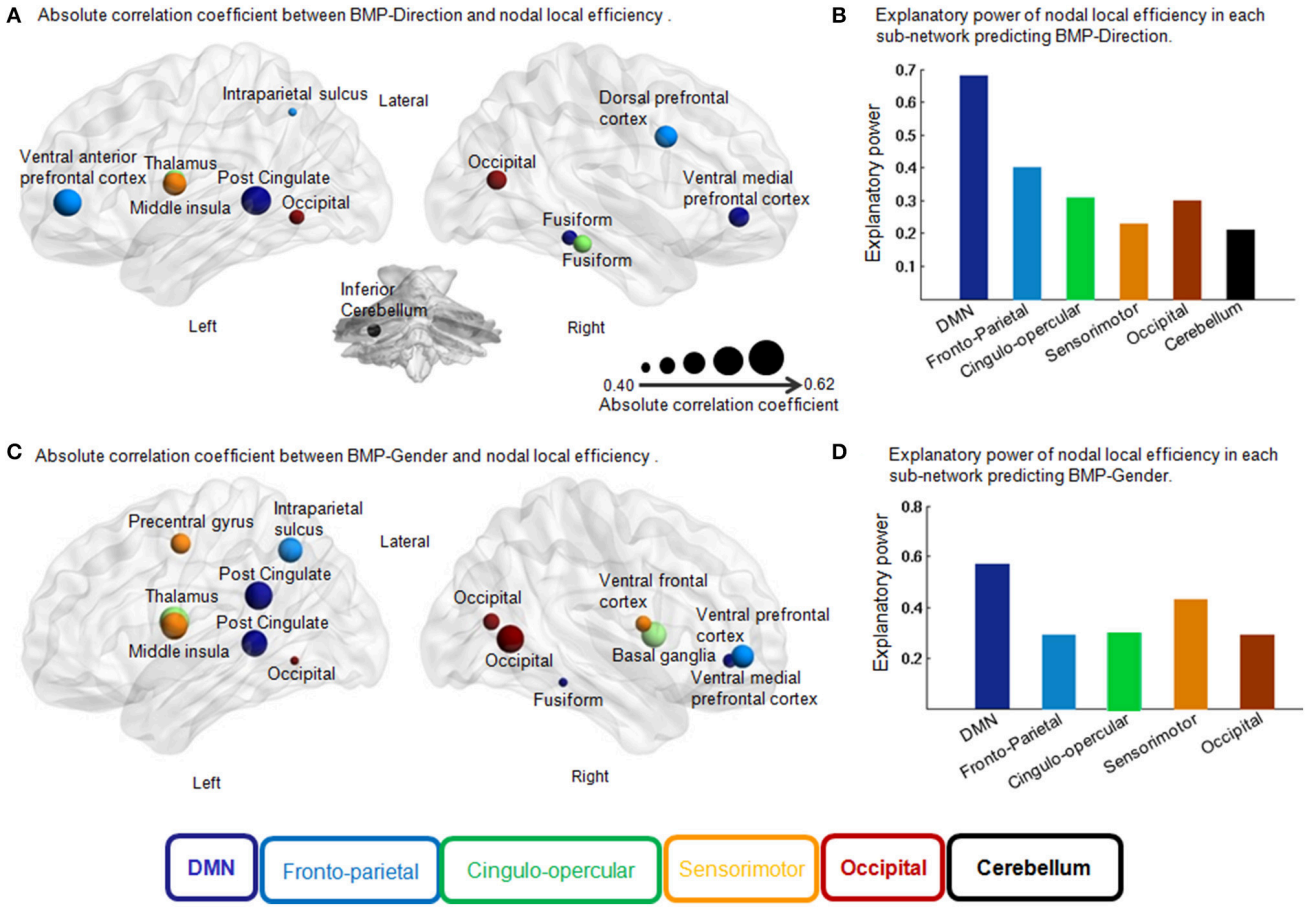
**Correlation coefficient of each region in the feature selection and independent explanatory power of each sub-network in the multiple linear regression models for BMP. (A)** Absolute correlation coefficient between BMP-Direction and nodal local efficiency. Pearson correlation, *p* < 0.05, uncorrected. The size of each region indicates the absolute correlation coefficient. The different colors of the regions indicate different sub-networks. **(B)** Explanatory power of nodal local efficiency in each sub-network predicting BMP-Direction. Fronto-parietal sub-network: *F*_(3, 20)_ = 4.39, *p* = 0.02, *R*^2^ = 0.40; cingulo-opercular sub-network: *F*_(2, 21)_ = 4.75, *p* = 0.02, *R*^2^ = 0.31; occipital sub-network: *F*_(1, 21)_ = 4.55, *p* = 0.02, *R*^2^ = 0.30; sensorimotor sub-network: *F*_(1, 22)_ = 6.56, *p* = 0.02, *R*^2^ = 0.23 and cerebellar sub-network: *F*_(1, 22)_ = 5.70, *p* = 0.03, *R*^2^ = 0.21. **(C)** Absolute correlation coefficient between BMP-Gender and nodal local efficiency. Pearson correlation, *p* < 0.05, uncorrected. The size of each region indicates the absolute correlation coefficient. **(D)** Explanatory power of nodal local efficiency in each sub-network predicting BMP-Gender. Sensorimotor sub-network: *F*_(3, 20)_ = 5.02, *p* = 0.01, *R*^2^ = 0.43; cingulo-opercular sub-network: *F*_(2, 21)_ = 4.56, *p* = 0.02, *R*^2^ = 0.30 and front-parietal sub-network: *F*_(2, 21)_ = 4.24, *p* = 0.03, *R*^2^ = 0.29. Abbreviations: DMN, Default Mode Network; BMP, biological motion perception; BMP-Direction, direction perception of biological motion; BMP-Gender, gender perception of biological motion.

We found that the nodal local efficiency of some regions showed significant correlations with IE index of BMP performance (for details see Table 2 and Figure 5). These regions were predominately distributed across the default mode network (DMN), cingulo-opercular, fronto-parietal and vision-related networks (Table 2). The nodal local efficiency of these regions from each participant was showed in Figure 3. We also performed multiple linear regression analysis to evaluate the contributions of all these regions in predicting BMP performance. We found that the nodal local efficiency of all these regions could explain 88% of the inter-individual variability of BMP-Direction [*F*_(12, 11)_ = 6.52, *p* = 1.99e-3, *R*^2^ = 0.88] and 91% of BMP-Gender [*F*_(14, 9)_ = 6.85, *p* = 3.26e-3, *R*^2^ = 0.91]. Despite this, the regression coefficient between the nodal local efficiency of each region with the IE index of BMP-Direction and BMP-Gender performance did not survive the threshold of *p* < 0.05.

**Table 2:** **Regions of nodal local and global efficiency showed significant correlation with inverse efficiency of biological motion perception (BMP)**.

	**Peak MNI coordinates**				**Pearson correlation coefficients**				**Regression coefficients**			
**Brain regions**	***X***	***Y***	***Z***	**Sub−network**	***BD*^a^**	***BG*^a^**	***BD*^b^**	***BG*^b^**	***BD*^a^**	***BG*^a^**	***BD*^b^**	***BG*^b^**
vmPFC_R	8	42	−5	Default	−0.54	−0.53^**^	−	−	−0.40^**^	−0.37^*^	−	−
PCC_R	10	−55	17	Default	−	−	−0.46^*^	−	−	−	−0.07	
PCC_L	−11	−58	17	Default	−	−	−0.46^*^	−0.50^*^	−	−	−0.24	−0.50^*^
PCC_L	−5	−43	25	Default	−	−0.51^*^	−	−	−	−0.36^*^	−	−
PCC_L	−8	−41	3	Default	−0.62^**^	−0.49^*^	−	−	−0.42^**^	−0.34^*^	−	−
Fusiform_R	28	−37	−15	Default	−0.54^**^	−0.41^*^	−0.55^**^	−	−0.37^*^	−	−0.42	
vent aPFC_L	−43	47	2	Fronto-parietal	0.44^*^	−	−	−	0.32	−	−	−
vent aPFC_R	42	48	−3	Fronto-parietal	−	0.44^*^	−	−	−	0.34	−	−
vlPFC_R	39	42	16	Fronto-parietal	−	−	−	0.45^*^	−	−	−	0.32
dFC_L	44	8	34	Fronto-parietal	0.47^*^	−	−	−	0.3	−	−	
dlPFC_L	−44	27	33	Fronto-parietal	−	−	0.42^*^	−	−	−	0.35	
IPL_R	54	−44	43	Fronto-parietal	−	−	−	0.42^*^	−	−	0.37	0.25
IPS_L	−32	−58	46	Fronto-parietal	0.42^*^	0.43^*^	−	−	0.27	0.33	−	−
Thalamus_L	−12	−3	13	Cingulo-opercular	−0.51^*^	−0.55^**^	−	−	−0.37	−0.1	−	−
Fusiform_R	54	−31	−18	Cingulo-opercular	0.46^*^	−	−	−	0.27		−	−
Basal ganglia_R	14	6	7	Cingulo-opercular	−	−0.46^*^	−	−	−	−0.47	−	−
TPJ_L	−52	−63	15	Cingulo-opercular	−	−	−0.42^*^	−	−	−	−0.42^*^	
Middle insula_R	37	−2	−3	Cingulo-opercular	−	−	−	0.49^*^	−	−	−	0.49^*^
vFC_R	43	1	12	Sensorimotor	−	0.46^*^	−	−	−	0.24	−	−
Middle insula_L	−42	−3	11	Sensorimotor	0.48^*^	0.53^**^	−	−	0.48^*^	0.42^*^	−	−
Precentral gyrus_L	−44	−6	49	Sensorimotor	−	0.45^*^	−	−	−	0.22	−	−
Parietal_L	−38	−15	59	Sensorimotor	−	−	−0.48^*^	−0.43^*^	−	−	−0.39^*^	−0.41
Precentral gyrus_L	−54	−22	22	Sensorimotor	−	−	−0.41^*^	−	−	−	−0.31	
Parietal_L	−24	−30	64	Sensorimotor	−	−	−	0.42^*^	−	−	−	0.41
Occipital_L	−34	−60	−5	Occipital	−0.43^*^	−0.47^*^	−	−	−0.21	−0.30	−	−
Occipital_R	39	−71	13	Occipital	−0.52^**^	−0.47^*^	−	−	−0.41	−0.30	−	−
Temporal_R	46	−62	5	Occipital	−	−0.41^*^	−	−	−	−0.02	−	−
Occipital_L	−16	−76	33	Occipital	−	−	−0.44^*^	−	−	−	−0.44^**^	−
Inferior cerebellum_L	−25	−60	−34	Cerebellum	0.45^*^	−	−	−	0.45^*^	−	−	−

a *Nodal local efficiency*.

b Nodal global efficiency; BD, direction perception of biological motion; BG, gender perception of biological motion; R, right hemisphere; L, left hemisphere. Pearson correlation coefficients:

** *p <0.01*,

* p <0.05, uncorrected. Regression coefficients: ^**^p <0.01, ^*^p <0.05, FDR corrected. vmPFC, ventral medial prefrontal cortex; PCC, post-cingulate cortex; vent aPFC, ventral anterior prefrontal cortex; vlPFC, ventral lateral prefrontal cortex; dFC, dorsal prefrontal cortex; dlPFC, dorsolateral prefrontal cortex; IPL, inferior parietal lobe; IPS, intra−parietal sulcus; TPJ, temporoparietal junction; vFC, ventral frontal cortex.

Next we assessed the contributions of the regions that belonged to the same sub-network in BMP performance predictions using linear regression models. The regression coefficients between sub-network regions and IE indices of BMP performance were also shown in Table 2. Of these sub-networks, the nodal local efficiency of each region in DMN showed significant regression coefficient with BMP-Direction and BMP-Gender performance. These regression coefficients also survived after false discovery rate (FDR) correction, *p* < 0.05. The nodal local efficiency of regions in DMN explained 68% of the inter-individual variance of BMP-Direction [*F*_(3, 20)_ = 13.85, *p* < 0.0001, *R*^2^ = 0.68] and 57% of the variance of BMP-Gender [*F*_(4, 19)_ = 6.40, *p* = 1.94e−3, *R*^2^ = 0.57]. Such high explanatory power was not observed in the other sub-networks (Figure 5B).

Regarding nodal global efficiency, a number of regions in DMN, fronto-parietal network, cingulo-opercular network, sensorimotor, and vision-related sub-networks exhibited significant correlations between nodal global efficiency with IE indices of BMP-Direction and BMP-Gender performance (Table 2). The nodal global efficiency of these regions could predict 60% of the inter-individual variability of BMP-Gender performance [*F*_(6, 17)_ = 4.17, *p* = 0.02, *R*^2^ = 0.60] but failed to predict the variability in BMP-Direction performance [*F*_(9, 14)_ = 2.37, *p* = 0.07]. In addition, all the regression coefficients of these regions did not survive at the threshold of *p* < 0.05.

However, among these sub-networks, the nodal global efficiency of regions in DMN explained 38% of BMP-Direction [*F*_(3, 20)_ = 4.04, *p* = 0.02, *R*^2^ = 0.38] and 25% of BMP-Gender [*F*_(1, 22)_ = 7.17, *p* = 0.01, *R*^2^ = 0.25]. The regression coefficients between nodal global efficiency of each region in DMN and BMP-Gender performance survived after FDR correction (*p* < 0.05) and are shown in Table 2.

#### Static direction and gender perception

In the feature selection, we found that the nodal local and global efficiency of several regions were significantly correlated with IE indices of static direction and gender perception performance (Table 3). Using a multiple regression model, we found the inter-individual variability of IE indices of static direction and gender information perception performance could be effectively explained by nodal global efficiency of the related regions [i.e., Static-Direction perception: *F*_(9, 6)_ = 11.56, *p* = 3.76e-3, *R*^2^ = 0.95; Static-Gender perception: *F*_(12, 3)_ = 20.06, *p* = 0.02, *R*^2^ = 0.98]. However, none of the regression coefficients of nodal local and global efficiency of these regions reached the threshold of *p* < 0.05. In the regression model based on sub-networks, the nodal global efficiency of regions in the fronto-parietal sub-network had the highest explanatory power and explained 71% of IE index of Static-Gender information perception [*F*_(3, 10)_ = 10.20, *p* = 1.28e−3, *R*^2^ = 0.71] and 71% of Static-Direction information perception [*F*_(2, 13)_ = 16.03, *p* = 3.09e−4, *R*^2^ = 0.71].

**Table 3:** **Regions of nodal local and global efficiency showed significant correlation with inverse efficiency of static gender and direction perception**.

	**Peak MNI coordinates**				**Pearson correlation coefficients**				**Regression coefficients**			
**Brain regions**	***x***	***y***	***z***	**Sub−network**	***SD*^a^**	***SG*^a^**	***SD*^b^**	***SG*^b^**	***SD*^a^**	***SG*^a^**	***SD*^b^**	***SG*^b^**
vmPFC_L	−11	45	17	Default	−	0.66^**^	−	−	−	0.66^**^	−	−
vlPFC_R	46	39	−15	Default	−	−	−	0.50^*^	−	−	−	0.18
Sup frontal_L	−16	29	54	Default	−	−	−	0.56^*^	−	−	−	−0.22
Fusiform_R	28	−37	−15	Default	−	−	−	−0.57^*^	−	−	−	−0.68^**^
Precuneus _L	−6	−56	29	Default	−	−	−	0.54^*^	−	−	−	0.27
Inf temporal_L	−59	−25	−15	Default	−	−	0.52^*^	0.51^*^	−	−	0.52^*^	0.56^*^
aPFC_L	−29	57	10	Fronto-parietal	−	0.70^**^	−	−	−	0.70^**^	−	−
vent aPFC_L	−43	47	2	Fronto-parietal	−	−	0.77^**^	0.75^**^	−	−	0.48^*^	0.39
dlPFC_L	−44	27	33	Fronto-parietal	−	−	0.76^**^	0.76^**^	−	−	0.45^*^	0.46^*^
IPL_L	−48	−47	49	Fronto-parietal	−	−	−	0.54^*^	−	−	−	0.15
vPFC_R	34	32	7	Cingulo-opercular	−	−	0.51^*^	−	−	−	0.51^*^	−
vFC_L	−48	6	1	Cingulo-opercular	0.53^*^	−	−	−	0.17	−	−	−
vFC_L	−46	10	14	Cingulo-opercular	−	0.50^*^	−	−	−	0.50^*^	−	−
Mid insula_L	−30	−14	1	Cingulo-opercular	−	−	−	−0.53^*^	−	−	−	−0.53^*^
TPJ_L	−52	−63	15	Cingulo-opercular	0.55^*^	−	−	−	0.27	−	−	−
Parietal_R	58	−41	20	Cingulo-opercular	0.51^*^	−	−	−	0.26	−	−	−
vFC_R	43	1	12	Sensorimotor	0.63^**^	−	−	−	−0.33	−	−	−
Middle insula_L	−42	−3	11	Sensorimotor	0.53^*^	−	−	−	0.39	−		−
Temporal_L	−41	−37	16	Sensorimotor	−	0.53^*^	−0.51^*^	−0.73^**^	−	0.43^*^	−0.53^*^	−0.73^**^
Precentral gyrus_L	−54	−9	23	Sensorimotor	−	−	−0.52^*^	−	−	−	−0.51^*^	−
Temporal_L	−54	−22	9	Sensorimotor	−	−0.61^*^	−	−	−	−0.54^*^	−	−
Occipital_L	−34	−60	−5	Occipital	−	−	−0.74^**^	−0.61^*^	−	−	−0.56	−0.42
Occipital_R	15	−77	32	Occipital	−	−	−0.60^*^	−	−	−	−0.06	−
Occipital_R	39	−71	13	Occipital	−0.57^*^	0.63^**^	−0.50^*^	−0.57^*^	−0.72	−1.39^*^	−0.24	−0.29
Occipital_L	−34	−60	−5	Occipital	−0.74^**^	−0.53^*^	−	−	−0.81	−0.87^*^	−	−
Post-occipital_L	−37	−83	−2	Occipital	−0.53^*^	−0.62^*^	−	−	−0.04	0.51	−	−
Post-occipital_L	−29	−88	8	Occipital	−0.50^*^	−0.57^*^	−	−	−0.08	−1.02	−	−
Occipital_R	19	−66	−1	Occipital	−0.74^**^	−0.59^*^	−	−	−0.79	−1.54^*^	−	−
Post-occipital_R	33	−81	−2	Occipital	−0.67^**^	−0.51^*^	−	−	0.58	1.58^*^	−	−
Occipital _R	36	−60	−8	Occipital	−0.71^**^	−0.60^*^	−	−	0.68	1.46^*^	−	−

a *Nodal local efficiency*.

b Nodal global efficiency; SD, static direction information, SG, static gender information. Pearson correlation coefficients:

** *p <0.01*,

* p <0.05, uncorrected. Regression coefficients: ^**^p <0.01, ^*^p <0.05, FDR corrected. Inf temporal, inferior temporal; Sup frontal, superior frontal cortex; vlPFC, ventrolateral prefrontal cortex; vmPFC, ventral medial prefrontal cortex; dlPFC, dorsolateral prefrontal cortex; vent aPFC, ventral anterior prefrontal cortex; IPL, inferior parietal lobe; aPFC, anterior prefrontal cortex; vPFC, ventral prefrontal cortex; vFC, ventral frontal cortex; TPJ, temporoparietal junction; Post-occipital, posterior occipital.

The nodal local efficiency of all the correlated regions effectively explained the IE index of Static-Direction performance [*F*_(12, 3)_ = 10.79, *p* = 0.04, *R*^2^ = 0.98] and had a significant trend explaining the Static-Gender performance [*F*_(12, 3)_ = 8.69, *p* = 0.05]. In the regression model based on sub-networks, we found that the nodal local efficiency of regions in occipital sub-network explained 84% of the variance in Static-Gender perception [*F*_(7, 8)_ = 6.02, *p* = 0.01, *R*^2^ = 0.84] and 77% of variance in Static-Direction perception [*F*_(7, 8)_ = 3.87, *p* = 0.04, *R*^2^ = 0.77]. The regression coefficients of the nodal global and local efficiency of each region based on sub-networks with static information perception are shown in Table 3.

#### Discrimination analysis

**Figure 6:**
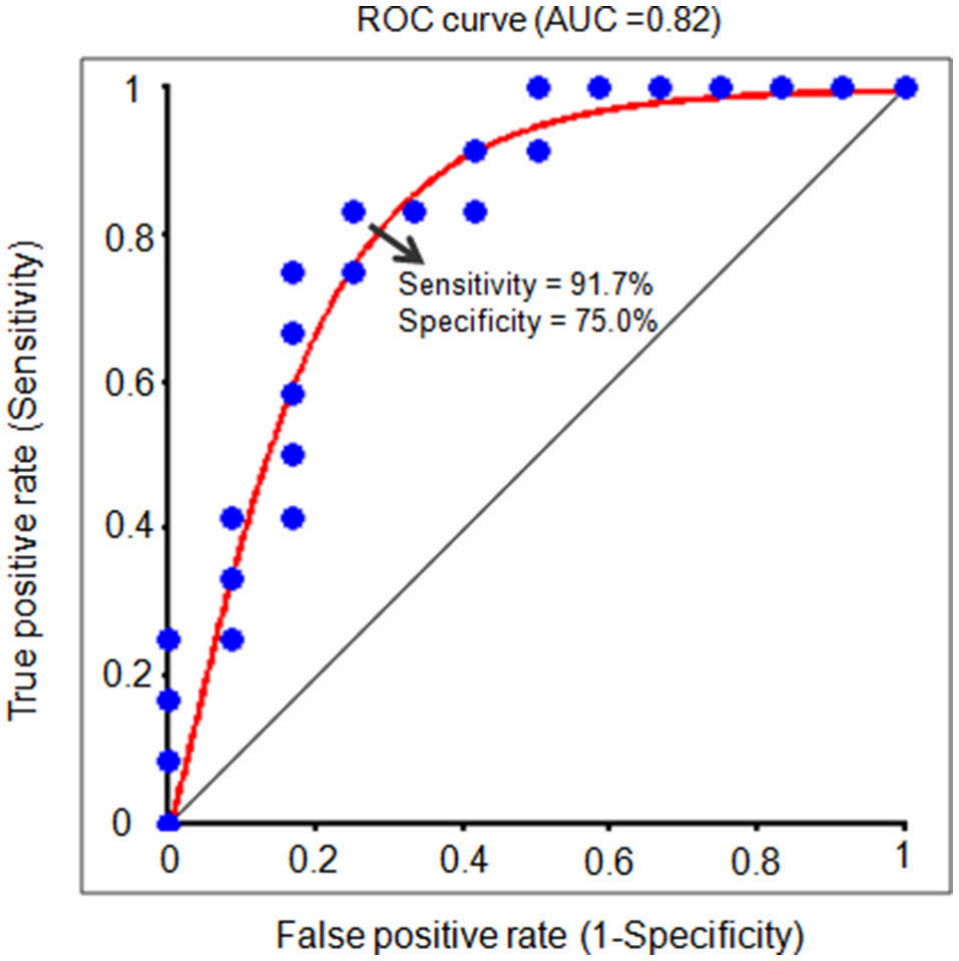
**The receiver operating characteristic (ROC) curve for distinguishing the IE index for each individual**. The ROC curve indicates a function of nodal local efficiency of the left thalamus in dividing high and low IE groups. A high area under the ROC (AUC) means a higher probability that a randomly chosen high IE group example is ranked higher than a randomly chosen low IE example. The maximum sensitivity (the proportion of high IE group that are correctly identified as high group) of the ROC is 91.7% and a specificity (the proportion of low IE group that are correctly identified as low group) of 75.0%. IE, inverse efficiency, the average response time of correct trials was divided by the related accuracy ratio.

In order to further test the association between the nodal local and global efficiency, and the BMP performance, we divided all the participants into high and low IE index groups according to their median IE values. Based on this group-difference, a ROC analysis was used to explore whether the nodal properties of the above brain network could effectively discriminate the two groups. There were some regions whose nodal local efficiencies could effectively be used to discriminate the high and low IE groups using ROC analysis (Table 4). Figure 6 presents the ROC of nodal local efficiency of the left thalamus in the two group's discrimination (AUC = 0.82, *p* = 0.01, 95% CI area = 0.65–0.99) with a maximum sensitivity of 91.7% and a specificity of 75.0%. However, the nodal global efficiency was not able to be used to discriminate the high and low IE groups.

**Table 4:** **ROC classification of high and low inverse efficiency groups of biological motion perception based on nodal local efficiency**.

	**Peak MNI coordinates**				**Classification**			
**Brain regions**	***x***	***y***	***z***	**Hemisphere**	**AUC^a^**	***p*^a^**	**AUC^b^**	***p*^b^**
Post-cingulate cortex	−8	−41	3	L	0.74	0.03	0.74	0.02
IPS	−32	−58	46	L	0.75	0.02	0.75	0.03
Thalamus cortex	−12	−3	13	L	0.82	0.01	−	−
Fusiform gyrus	54	−31	−18	R	0.78	0.01	−	−
vaPFC	42	48	−3	R	−	−	0.78	0.03
Occipital lobe	−34	−60	−5	L	0.76	0.03	0.73	0.04

a *Direction perception of biological motion*.

b *Gender perception of biological motion; R, right hemisphere; L, left hemisphere. Abbreviations: ROC, receiver operating characteristic; AUC, area under the ROC; IPS, intra-parietal sulcus; vaPFC, ventral anterior prefrontal cortex*.

### Validation analysis findings

In order to validate the results of the multiple linear regression analysis between the network regional efficiency and BMP performance, the IE indices of BMP across participants were shuffled to disorganize their correspondences with the network properties. We found that significantly correlated regions could no longer explain the BMP inter-individual variability in nodal local efficiency [BMP-Direction: *F*_(12, 11)_ = 1.11, *p* = 0.45; BMP-Gender: *F*_(14, 9)_ = 1.15, *p* = 0.42] or nodal global efficiency [BMP-Direction: *F*_(9, 14)_ = 0.27, *p* = 0.96; BMP-Gender: *F*_(6, 17)_ = 0.99, *p* = 0.47].

We also found that the nodal efficiency of the regions without significant correlations (Pearson correlation, *p* > 0.05) with IE index of BMP could not effectively explain BMP performance in nodal local efficiency [BMP-Direction: *F*_(12, 11)_ = 0.56, *p* = 0.84; BMP-Gender: *F*_(14, 9)_ = 0.59; *p* = 0.75] or nodal global efficiency [BMP-Direction: *F*_(9, 14)_ = 1.88, *p* = 0.16; BMP-Gender: *F*_(6, 17)_ = 0.79, *p* = 0.61].

The functional weighted brain network related to AAL-90 brain template was also small-world organized (Figure 4). The results showed that nodal local efficiency (Table S1) could effectively predict the participants' IE indices of BMP performance [BMP-Direction: *F*_(6, 17)_ = 7.24, *p* < 0.0001, *R*^2^ = 0.72; BMP-Gender: *F*_(4, 19)_ = 7.54, *p* < 0.0001, *R*^2^ = 0.61]. The key regions with high explanatory power were also located at the DMN modules (e.g., BMP-Direction: anterior cingulate and post-cingulate gyrus; BMP-Gender: the orbital and medial parts of the superior frontal gyrus). By contrast, nodal global efficiency failed to explain either BMP-Direction [*F*_(8, 15)_ = 2.57, *p* = 0.05] or BMP-Gender [*F*_(9, 14)_ = 1.95, *p* = 0.13]. For the perception of static information, nodal global efficiency could effectively predict static information perception [Static-Direction: *F*_(11, 4)_ = 7.45, *p* = 0.03, *R*^2^ = 0.95; Static-Gender, *F*_(9, 6)_ = 4.31, *p* = 0.04, *R*^2^ = 0.86], whereas nodal local efficiency could effectively explain Static-Direction [*F*_(7, 8)_ = 6.67, *p* = 7.89e-3, *R*^2^ = 0.85] but failed to explain Static-Gender [*F*_(9, 6)_ = 2.72, *p* = 0.14].

## Discussion

The present study explored the association between intrinsic functional brain network activity and BMP performance. The main findings can be summarized as follows: (i) there exist linear associations between intrinsic brain network efficiencies and BMP performance, which could be measured using a multiple linear regression model; (ii) the nodal local efficiency rather than the nodal global efficiency could account for individual variance in BMP performance; and (iii) the nodal local efficiencies of the DMN explain most of the variation in BMP performance.

As a central component of our perception system, BMP assists in interaction with the dynamic natural environment (Beauchamp et al., 2003; Kilts et al., 2003; Peelen et al., 2006). BMP processes are dissociated from processes of static form perception (Hiris, 2007; Thirkettle et al., 2009; Roché et al., 2013; Fraiman et al., 2014). Many previous studies have shown that observers can correctly identify the gender and direction (Mather and Murdoch, 1994; Troje, 2002; Pollick et al., 2005; Blake and Shiffrar, 2007) of a walking figure based on dynamic cues in biological motion presented by point-light displays. Even though the detection of motion is an intrinsic capacity of the visual system, the perception of motion varies across individuals (Langer et al., 2011; Sala-Llonch et al., 2012; Stevens et al., 2012; Pamplona et al., 2015a). However, few studies have explored the neural substrates underlying inter-individual variability in BMP performance (Gilaie-Dotan et al., 2013; Pavlova et al., 2014).

In recent years, intrinsic brain activity, especially the properties of the brain network, was widely used to reveal the underlying mechanism of mental abilities and mental states (Latora and Marchiori, 2001; Lewis et al., 2014). The concept of network efficiency was used to measure how efficiently information is exchanged within a network (Latora and Marchiori, 2001) both on a global and local scale. The global efficiency of the network is the efficiency of a parallel system, where all the nodes in a network concurrently exchange packets of information. On the other hand, the local efficiency of a network reveals how much the system is fault tolerant, showing how efficient the communication across the nearest neighbors of one node is when this node is removed (Latora and Marchiori, 2001; Bullmore and Sporns, 2009; Rubinov and Sporns, 2010). A small-worldness network always has high global and local efficiency (Xue et al., 2011). In our study, we found that the global and local efficiencies of the network are not significantly correlated with individual BMP, which indicates that the inter-variability of BMP may not be predicted by the overall global and local efficiencies of the network.

Nodal global and local efficiencies measure the extent to which each node connects all other nodes of a network, and how well information propagates through the network (Xue et al., 2011; Lewis et al., 2014; Zhang et al., 2015). Using Pearson correlation analysis, we found that nodal global and local efficiencies of some regions showed significant correlations with BMP and static pictures perception (Tables 2, 3). In previous studies (Schilbach et al., 2008; Laird et al., 2011; Zhang et al., 2015) both the nodal global and local efficiencies have been proven to be linked with neurodegenerative disease and cognitive abilities. For example, in our recent study, Zhang et al. (2015) found that the network nodal local efficiency could effectively discriminate Parkinson's disease patients from healthy controls using multivariate pattern analysis, and could also describe the variability of tremor based on a multiple linear regression model. In another previous study, Pamplona et al. (2015b) found that the nodal local efficiency was associated with verbal comprehension ability.

Notably, using multiple regression analysis, we found that the nodal local and global efficiencies performed differently in predicting social information perception performance from biological motion and static pictures (Tables 2, 3). The nodal local efficiency explained most of the inter-variability of BMP, while the nodal global efficiency predicted the performance of static pictures perception. These observations were highly independent of network type (i.e., networks constructed by Dosenbach-160 or AAL-90). The Dosenbach-160 brain template include 160 spherical regions that were generated based on a meta-analysis (Dosenbach et al., 2010) which were divided into six sub-networks: cingulo-opercular, fronto-parietal, default mode, sensorimotor, occipital, and cerebellar. The AAL-90 atlas is a widely used manual macroanatomical parcellation of the single subject MNI-space template brain (Tzourio-Mazoyer et al., 2002). In our study, the functional networks constructed by Dosenbach-160 and AAL-90 brain templates were both small-worldness organized (Figure 4). In the validation analysis, we also found that those regions that did not correlate with BMP performance could not explain BMP performance. The regression model failed to explain BMP performance when the consistency between network efficiency and BMP performance was shuffled. Despite the brain-behavior association analysis, in the group-discrimination analysis we also found that the nodal local efficiency instead of the nodal global efficiency could discriminate the high and low perception groups. All of these above findings suggest that there might be a local network efficiency pattern that is functionally associated with individual BMP performance. By contrast, we found that nodal global network efficiency predicted the static direction and gender information perception on functional networks constructed on Dosenbach-160 and AAL-90 brain templates. Thus, these findings may collectively suggest the existence of a specific pattern of connections between individual regions and adjacent regions within functional brain networks to assist BMP processes.

In addition, the independent contribution of each sub-network was further explored in explaining the inter-individual variability of BMP. Our results showed the importance of DMN in predicting BMP performance. Previous studies have demonstrated the involvement of the DMN in many types of social-cognitive functions (Northoff et al., 2010; Mantini and Vanduffel, 2013), such as self-referential processing (Frith and Frith, 2006; Mars et al., 2012), mentalizing and theory of mind (Raichle et al., 2001; Greicius et al., 2003; Raichle and Snyder, 2007; Buckner et al., 2008). In our study, we found that the DMN nodes were distributed across the posterior cingulate cortex, ventral medial prefrontal cortex, and fusiform gyrus. These regions largely overlap with the DMN regions identified in previous studies (Blakemore and Decety, 2001; Pelphrey et al., 2004; Kourtzi et al., 2008). It is very important for people to understand other's actions and infer their goals or intentions during social interaction (Van Overwalle and Baetens, 2009; Centelles et al., 2011). Our findings provide evidence for a close association between spontaneous neural activity of the DMN and BMP.

Different from BMP, we found that nodal global efficiency of the fronto-parietal network had a high estimation to predict inter-individual differences of static information perception. In our study, the regions of the fronto-parietal network included the dorsolateral prefrontal cortex and anterior prefrontal cortex. In previous studies (Miller and Cohen, 2001; O'Reilly, 2006, 2010), the fronto-parietal network has long been implicated as a source of attentional control. The prefrontal cortex is known to be important for cognitive control, enabling behavior to be at once flexible yet task-focused. Therefore, in our study, the nodal global efficiency of the fronto-parietal network could predict static information perception, which may indicate that attention and cognitive control play important role in gender and direction perception of static information.

## Conclusion

In conclusion, the present study measured inter-individual variability of BMP and spatial organization of intrinsic brain networks. We found that the nodal local efficiency could explain BMP performance using a multiple linear regression model in which the DMN played an important role. These findings suggest that the information translation ability of the local circuit (e.g., DMN) of the intrinsic brain network may be the neural basis of BMP performance. This study will help identify the underlying neural mechanisms of BMP.

## Author contributions

ZW and DZ had full access to all the data in the study and takes responsibility for the integrity of the data and the accuracy of the data analysis. Study concept and design: ZW, DZ, RH, and ML. Acqusition, analysis or interpretation of data: ZW, SC, and JP. Drafting of the manuscript: ZW, DZ, RH, and ML. Critical revision of the manuscript for important intellectual content: ZW, DZ, BL, RH, and ML. Statistical analysis, ZW, DZ, SC, and JP. Obtained funding: ZW, BL, SC. Administrative, technical, or material support: ZW, DZ, RH, and ML. Study supervision: ZW, DZ, RH, and ML.

### Conflict of interest statement

The authors declare that the research was conducted in the absence of any commercial or financial relationships that could be construed as a potential conflict of interest.
