# Supplementary material for: Prediction of Biological Motion Perception Performance from Intrinsic Brain Network Regional Efficiency
Source: Front Hum Neurosci. 2016 Nov 2;10:552. doi: 10.3389/fnhum.2016.00552 (PMC5090005; doi:10.3389/fnhum.2016.00552)
Supplement: Supplementary file 1 [file Table1.DOC]

Table S1. Regions of nodal local and global efficiency showed significant correlation with gender and direction biological motion perception.

|  |  |  | Pearson Correlation Coefficients | |
| --- | --- | --- | --- | --- |
| Nodal Efficiency | Brain Regions | Sub-network | BD | BG |
| $E_{loc}^{nodal}$ | IFGtriang.L | Prefrontal | 0.50* | 0.42* |
|  | ACG.R | Prefrontal | -0.52* | - |
|  | AMYG.R | Temporal | -0.6** | -0.57** |
|  | MOG.R | Occipital | -0.54** | - |
|  | PoCG.L | Parietal | 0.42* | - |
|  | SMG.R | Parietal | 0.53** | 0.58** |
|  |  |  |  |  |
| $E_{glob}^{nodal}$ | ORBsup.L | Prefrontal | 0.51* | 0.48* |
|  | ORBmid.L | Prefrontal | 0.44* | 0.41* |
|  | SFGmed.R | Prefrontal | -0.44* | - |
|  | INS.R | Subcortical | - | 0.45* |
|  | HIP.L | Temporal | -0.49* | -0.53** |
|  | HIP.R | Temporal | -0.58** | -0.63** |
|  | PHG.L | Temporal | -0.44* | -0.46* |
|  | PHG.R | Temporal | -0.60** | -0.59** |
|  | FFG.L | Temporal | - | -0.41* |
|  | SMG.L | Parietal | 0.53** | 0.53** |

Note: The network was constructed based on AAL-90 brain template. Pearson correlation coefficient: * *p* < 0.05,** *p* < 0.01, uncorrected. $E_{loc}^{nodal}$, nodal local efficiency; $E_{glob}^{nodal}$, nodal global efficiency. BD, direction perception of biological motion; BG, gender perception of biological motion. IFGtriang, inferior frontal gyrus, triangular part; ACG, anterior cingulate and paracingualte gyri; AMYG, amygdala; MOG, middle occipital gyrus; PoCG, postcentral gyrus; SMG, supramarginal gyrus; ORBsup, superior frontal gyrus, orbital part; ORBmid, middle frontal gryus, orbital part; SFGmed, superior frontal gyrus, medial part; INS, insula; HIP, hippocampus; PHG, parahippocampal gyrus; FFG, fusiform gyrus.
